# Neural Matrix Factorization Recommendation for User Preference Prediction Based on Explicit and Implicit Feedback

**DOI:** 10.1155/2022/9593957

**Published:** 2022-01-10

**Authors:** Huazhen Liu, Wei Wang, Yihan Zhang, Renqian Gu, Yaqi Hao

**Affiliations:** ^1^School of Information & Electrical Engineering, Hebei University of Engineering, Handan 056038, China; ^2^Hebei Key Laboratory of Security & Protection Information Sensing and Processing, Hebei University of Engineering, Handan 056038, China; ^3^School of Internet of Things Engineering, Jiangnan University, Wuxi 214122, China

## Abstract

Explicit feedback and implicit feedback are two important types of heterogeneous data for constructing a recommendation system. The combination of the two can effectively improve the performance of the recommendation system. However, most of the current deep learning recommendation models fail to fully exploit the complementary advantages of two types of data combined and usually only use binary implicit feedback data. Thus, this paper proposes a neural matrix factorization recommendation algorithm (EINMF) based on explicit-implicit feedback. First, neural network is used to learn nonlinear feature of explicit-implicit feedback of user-item interaction. Second, combined with the traditional matrix factorization, explicit feedback is used to accurately reflect the explicit preference and the potential preferences of users to build a recommendation model; a new loss function is designed based on explicit-implicit feedback to obtain the best parameters through the neural network training to predict the preference of users for items; finally, according to prediction results, personalized recommendation list is pushed to the user. The feasibility, validity, and robustness are fully demonstrated in comparison with multiple baseline models on two real datasets.

## 1. Introduction

The rapid development of the Internet has caused information overload, and how to gain the required information from the massive data to provide users with accurate personalized services is an urgent problem for every Internet-related industry. Recommendation system is one of the effective methods to solve these problems, which can effectively improve the loyalty of website users and is a must-consider function for every website and APP today. Personalized recommendation systems generally construct recommendation models from the historical interaction data generated when users browse websites. The data are mainly divided into two types: explicit feedback data and implicit feedback data. Explicit feedback data are generally ratings, which can accurately reflect user preferences; implicit feedback data are primarily clicks, favorites, and other user browsing behaviors, which can be converted into binary data, although they cannot accurately distinguish the degree of user preferences but can reflect the user's potential interest preferences. The recommendation model constructed by simple user implicit feedback or explicit feedback has poor performance and cannot satisfy the demands of rich scenarios in real applications, while the recommendation model constructed by combining two types of heterogeneous feedback data, which are complementary, can effectively improve the recommendation quality [1], but most recommendation models are constructed by using only one type of data, and the complementary benefits of combining the two are not fully exploited.

The collaborative filtering (CF) algorithm is the most widely used personalized recommendation method [2–4], which is mainly divided into neighborhood-based approaches and model-based approaches. Neighbor-based approach such as Item-KNN [5] is the most widely applied in industry; it calculates the similarity between items based on user-item history interactions and then generates the list of the user's top-N recommended items accordingly. Although the neighborhood-based approach is highly interpretable, it is less scalable and flexible than the model-based recommendation approach and has low recommendation relevance. Model-based approaches mainly construct a user preference model to predict user-item latent preferences, among which matrix factorization (MF) recommendation is the most popular and effective model-based recommendation method [3, 6] with high scalability and low complexity. It uses latent factor vectors to characterize users and items, maps users and items to a joint low-dimensional latent space, and formulates the recommendation as a user preference prediction problem for items based on the inner product of the corresponding user and item latent factor vectors. Early applications of matrix factorization techniques in recommender systems mainly focused on explicit feedback rating prediction [6, 7], which utilizes explicit user ratings of items for user preference prediction. However, subsequent researchers have found that this approach of modeling only a few observed positive feedback data resulted in poor performance of realistic top-N recommendation systems [8]. Therefore, some researchers have subsequently proposed matrix factorization algorithms based on implicit feedback like user clicks and favorites [3, 9], such as BPR-MF [10], which learns from implicit feedback with paired ranking targets and uses Bayesian personalized ranking objective function for optimization of matrix factorization model parameters; but implicit feedback is usually binary positive sample data, missing negative samples data, and improper acquisition of negative feedback data will affect the performance of the recommendation system. The SVD-based recommendation model, SVD++ [11], combines explicit feedback and implicit feedback. First, obtaining the implicit user factor matrix from the implicit feedback data combines it with the explicit user factor matrix, and then the linear inner product of the user factor matrix and the explicit item factor matrix are used to predict users' preferences for items.

The traditional matrix factorization model uses the dot product of user and item vectors to model the linear interaction between users-items, which cannot model the complex nonlinear deep feature representation between users-items well [12]. In recent years, with the rapid development and application of deep learning technology, some researchers have proposed using deep learning-based recommendation methods to model complex nonlinear interactions between users-items and achieve high quality recommendation effects. However, most of the current deep learning-based recommendation algorithms are used for modeling user-item interactions based on one type of the explicit-implicit feedback data to predict the user's preferences. For example, the NCF model proposed by He et al. [13] uses the binary implicit feedback data to model the latent factor vectors of users and items in a nonlinear method using a multilayer perceptron (MLP) instead of the traditional linear inner product of matrix factorization to predict the user-item interactions' preferences through neural networks; the DeepFM model proposed by Guo et al. [14] uses FM to obtain the low-order user-item cross features while acquiring the higher-order representation of features and enhance the generalization ability of the model by combining higher-order features with lower-order features. The experimental results demonstrate the effectiveness of neural networks for recommender system performance improvement, but most deep learning recommendation models are built based on easily trained binary implicit feedback data, overlooking the effect of explicit rating data to accurately reflect users' interest preferences. Moreover, for other types of websites, such as movie, video, music, and other rating categories, explicit feedback data are more important than implicit feedback data for predicting user preferences in recommendation systems. Therefore, building a deep learning recommendation model combining explicit feedback has strong practical significance.

In view of the issues and deficiencies in the above work, we propose a neural matrix factorization user preference prediction algorithm suitable for explicit-implicit feedback, based on the concepts of implicit feedback-based deep matrix factorization model and traditional matrix factorization recommendation model, to make it adaptable to the evaluation classification scene, improve the accuracy of the recommendation system in predicting the user's preference for items, and show the personalized project recommendation list for users. In the algorithm, to utilize both explicit ratings and implicit feedback, this paper proposes a deep neural network latent factorization model for gaining deep feature representations of users and items. In the algorithm, using explicit and implicit feedback data as input, the user and item latent factor vectors are mapped to a nonlinear low-dimensional space. According to the matrix decomposition principle and the training of neural network, the best latent factor vectors of users and items are fitted to achieve user preference prediction. In the algorithm, a new loss function is also designed to fully consider both explicit feedback and implicit feedback to better optimize the parameters. Comparative experimental results demonstrate that the proposed algorithm can effectively model the nonlinear information of explicit feedback and implicit feedback of user-item interactions and improve the accuracy of the recommendation system in predicting user preferences (ratings). The main contributions of this paper are as follows:

1. A neural matrix factorization algorithm based on explicit-implicit feedback is EINMF, which learns the deep latent factor features of users and items, as well as the linear and nonlinear features of explicit-implicit feedback of user-item interactions, so that they are closely combined to jointly optimize the objective function and reach the goal of improving the accuracy of personalized recommendation
2. A new loss function is constructed which fully utilizes the information in the explicit feedback and implicit feedback from user interactions for the optimization and updating of model parameters
3. Based on the ranking evaluation (top-N), the recommendation performance of the EINMF algorithm is compared and analyzed with other baseline models for datasets of different sizes and sparsity, showing that EINMF always produces the best performance; in addition, the performances of the recommendation algorithm with different parameters are also compared and analyzed to prove its effectiveness and rationality

## 2. Related Work

### 2.1. Traditional Matrix Factorization Recommended Models

Matrix factorization is the most widely used model-based recommendation algorithm, which is popular among researchers for its scalability and fast prediction speed. Therefore, many enhanced MF algorithms based on matrix factorization have been deployed. The basic matrix factorization recommendation model mainly predicts users' preferences (ratings) for items by reducing the dimension of the original user-item rating matrix; that is, it uses the product of two low-dimensional dense matrix to represent the user-item explicit rating matrix approximately; it is later extended to a matrix factorization recommendation model based on implicit user-item feedback. The basic matrix factorization model recommendation model is generally represented as

(1) $$\begin{array}{c} R\approx U^{T}\cdot V. \end{array}$$

*R* represents the original or binary implicit feedback rating matrix of *m* users and *n* items, represented by the product of two low-rank dense matrices *U* ∈ *R*^*m*×*f*^ and *V* ∈ *R*^*n*×*f*^; *U* represents the user latent feature matrix, *V* represents the item latent feature matrix, and *f* represents the dimension of the user latent factor vector and the item latent factor vector after dimension reduction (*f* ≪ min{*m*, *n*}). The user preference prediction function *f*(*u*, *i*) is generally used to indicate preference of user *u* for item *i*, and the ratings of the user preference prediction obtained by regression building simulation are ${\hat{r}}_{ui}=f\left(u,i\right)$. Therefore, in matrix factorization model, a user's preference prediction score for an item can be expressed as

(2) $$\begin{array}{c} \mathrm{Prefercence}\left(u,i\right)={\hat{r}}_{ui}=f\left(u,i|p_{u},q_{i}\right)=p_{u}^{T}q_{i}=\overset{F}{\underset{f=1}{\sum }}p_{u,f}q_{f,i}. \end{array}$$

*p*_*u*_ represents the latent factor feature vector of user *u*, *q*_*i*_ represents the latent factor feature vector of item *i*, and rating *r*_*ui*_ by user *u* for item *i* is approximated by the dot product of the two vectors. At this point, there is an error between the predicted preference and the user's true preference: $e_{ui}=r_{ui}-{\hat{r}}_{ui}$; in order to get a more accurate predicted value of the user's preference, a matrix factorization recommendation model is thus established with a point-by-point loss function (objective optimization function), which is generally the square of the error:

(3) $$\begin{array}{c} L\left(r_{ui},p,q\right)=\underset{p^{*},q^{*}}{\min}\underset{\left(u,i\right)\in K}{\sum }{\left(r_{ui}-p_{u}^{T}q_{i}\right)}^{2}+\lambda \left({\left\|p_{u}\right\|}^{2}+{\left\|q_{i}\right\|}^{2}\right). \end{array}$$

In the above Formula, *K* denotes the set of user-item pairs with known true ratings in the training set, and the optimization of the objective function using stochastic gradient descent (SGD) or alternating least squares (ALS) [3, 6, 9, 11] can find the local minimum of the above objective function, so as to update the user latent feature vector *p*_*u*_ and the item latent feature vector *q*_*i*_ to obtain the optimum feature vector. The update formula is as follows, where *α* is the learning rate, which is used to control the update rate of the feature vector.

(4) $$\begin{array}{c} \left\{\begin{array}{c} p_{u,f}=p_{u,f}+\alpha \left(e_{ui}\cdot q_{f,i}-\lambda \cdot p_{u,f}\right)\\ q_{f,i}=q_{f,i}+\alpha \left(e_{ui}\cdot p_{u,f}-\lambda \cdot q_{f,i}\right) \end{array}\right.. \end{array}$$

In addition, Rendle et al. proposed a pairwise Bayesian personalized ranking learning method BPR-MF [10] based on implicit feedback and MF techniques, which treats top-N recommendations as a ranking issue and optimizes Bayesian pairwise ranking, the Maximum A Posteriori (MAP) estimation of users' pairwise preferences between interacted and noninteracted items, and is a sampling-based approach that uses a pairwise loss objective optimization function to optimize the model depending on the relative preferences of user-item pairs.

### 2.2. Deep Learning Recommended Models

Recently, due to the powerful representational learning capability, deep learning methods have been successfully applied in various fields, including computer vision, audio recognition, and natural language processing. Compared to traditional collaborative filtering algorithms, the application of deep learning in collaborative filtering algorithms has improved the richness of recommendations [12]. The deep learning collaborative filtering recommendation model takes the explicit rating feature vector or implicit feedback feature vector of users and items as the input of the neural network model, utilizes the deep learning model to learn the deep nonlinear features of users and items, similar to the matrix factorization recommendation model, constructs the objective optimization function with point-by-point loss or pairwise loss and so forth, learns and optimizes the best latent feature vectors of users and items, calculates the degree of user preference for items, and completes item recommendation.

In this paper, we utilize the neural collaborative filtering (NCF) [13] recommendation algorithm to construct a nonlinear neural network recommendation model, which exploits the nonlinear fitting ability of multilayer perceptron (MLP) to continuous functions to mine the explicit and implicit feedback data of user-item interactions and learn to gain explicit feedback user latent feature vectors and explicit feedback item latent feature vectors. NCF uses a multilayer perceptron machine to model bidirectional interactions between users and items, which aims to capture the nonlinear relationship between users and items, and its user preference prediction is defined as follows:

(5) $$\begin{array}{c} {\hat{y}}_{ui}=f\left(P^{T}v_{u}^{U},Q^{T}v_{i}^{I}|P,Q,\Theta_{f}\right), \end{array}$$

(6) $$\begin{array}{c} {\hat{y}}_{ui}=f\left(P^{T}v_{u}^{U},Q^{T}v_{i}^{I}\right)=\phi_{\mathrm{out}}\left(\phi_{X}\left(\ldots \phi_{2}\left(\phi_{1}\left(P^{T}v_{u}^{U},Q^{T}v_{i}^{I}\right)\right)\ldots \right)\right). \end{array}$$

However, most of the current deep learning collaborative filtering recommendation models construct recommendation models by implicit feedback data, such as the NCF [13] and ENMF [15] recommendation algorithms, which use multilayer perceptron (MLP) instead of dot product to learn latent feature vectors of users and items in implicit feedback. In contrast, ConvNCF [16] uses convolutional neural networks (CNNs) to learn higher-order correlations between user and item embedding dimensions based on implicit user-item interaction data. All of the above models utilize only the implicit feedback data of user-item interactions to obtain the fuzzy latent feature representations of users and items, ignoring the effects of explicit feedback to reflect users' precise preferences.

### 2.3. Explicit and Implicit Feedback for Recommendation

Many researchers have proposed model-based recommendation algorithms that simultaneously use both types of feedback data based on the respective characteristics of explicit and implicit feedback and the complementary advantages of combining the two. Koren fused explicit and implicit feedback data to obtain the item explicit factor matrix and implicit factor matrix, combined the item implicit factor matrix with the user explicit factor matrix as the user latent factor matrix, based on the matrix factorization algorithm, and proposed the SVD++ recommendation algorithm; Liu et al. considered the heterogeneity of explicit and implicit feedback, normalized the explicit ratings and binarized the implicit feedback, which mapped the data to the [0,1] interval uniformly, and proposed the matrix factorization model corating based on rating prediction [1]. Chen et al. proposed an EIFCF collaborative filtering recommendation algorithm [17], which processes implicit feedback data according to the weighted matrix factorization algorithm (GALS) to obtain latent implicit feature vectors of users and items, which are fused with explicit user and item latent feature vectors to jointly form user latent feature vectors and item latent feature vectors, to use explicit ratings and predict user preferences. Zhang et al. [18] established user and item feature matrices distinguishing positive feedback and negative feedback in explicit and implicit feedback data and then designed a novel rating prediction collaborative filtering recommendation algorithm, PNF-SVD++. Sun et al. proposed an EifSVD differential privacy collaborative filtering recommendation algorithm [19], according to the characteristics of the explicit-implicit feedback data, based on the SVD factorization recommendation algorithm, with the user-item rating matrix as input and the implicit features as a supplement to the explicit features, and adopted the gradient descent method to predict the user rating of item.

The studies of the above-mentioned explicit-implicit-feedback-based recommendation algorithms mostly use matrix factorization technique and its enhancement algorithm SVD++ as the base algorithm to learn the shallow linear features of users and items. On the other hand, the EINMF algorithm proposed in this paper combines the characteristics of explicit feedback data accurately reflecting users' preferences and implicit feedback data reflecting users' latent fuzzy preferences and utilizes matrix factorization algorithm to obtain shallow linear features and multilayer perceptron (MLP) to obtain deep nonlinear features of explicit and implicit feedback to construct a neural network matrix factorization user preference prediction recommendation model based on explicit and implicit feedback.

## 3. EINMF

### 3.1. Problem Formulation and Notation

In the EINMF model proposed in this paper, user-item ratings data are used as input to construct a recommendation model based on top-N recommendation. The numbers of users and items in the dataset are denoted using *m* and *n*. The set of users is *U*={*u*_1_, *u*_2_,…, *u*_*m*_}, and the set of items is *V*={*v*_1_, *v*_2_,…, *v*_*n*_}. According to the literature [13, 20], the known user ratings of items are marked as implicit feedback interactions as 1, and the unknown ratings are considered as implicit feedback and they are marked as 0. The task of top-N recommendation is to recommend a list with a set of items that are most interesting to a unique user in order to maximize the user satisfaction. When the top-N recommendation task is being conducted, the validity and accuracy of the recommendation are generally related to the final correct item ranking and less concerned with the exact rating [8]; therefore, all missing values in the user rating matrix are generally considered as 0, and the ratings are used to explicitly represent the different degrees of user preference for the items. Constructions of the user-item explicit rating matrix *R*=[*r*_*ui*_]_*m*×*n*_ and implicit feedback matrix *IR*=[*ir*_*ui*_]_*m*×*n*_ are shown in formulae (7) and (8). In this paper, we firstly construct a recommendation EINMF model with two types of feedback matrices to get the best user and item latent feature vectors; then, the user's preference for noninteracted items is predicted; finally, the noninteracted items are ranked according to their predicted preference values, the N items are got with the highest predicted preference values for the user, and they are recommended to the user in the form of a list or other forms to realize personalized recommendation.

(7) IRui=1,if interaction user u, item v is observed,0,un−interactionuser u, item v,

(8) $$\begin{array}{c} R_{ui}=\left\{\begin{array}{cc} r_{ui}, & \mathrm{if}\text{ rating}\left(\text{user }u\text{,item }v\right)\text{is observed,}\\ 0, & \text{rating is unknow}\left(\text{user }u\text{,item }v\right). \end{array}\right. \end{array}$$

The main symbols used in this paper are defined as shown in Table 1.

### 3.2. EINMF Model

**Figure 1:**
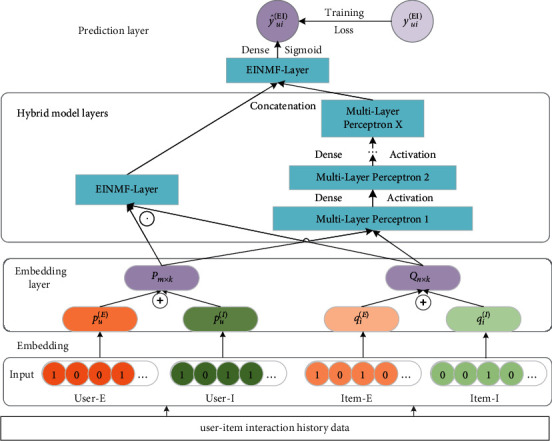
The structure of the EINMF model.

The design idea of the EINMF is as follows: the explicit rating matrix and the implicit feedback matrix of the users-items one-hot encoding processed by formulae (7) and (8) are used as the input of the neural matrix factorization model. Embedding is initialized with normal stochasticity to get the explicit-implicit feedback latent feature vectors *p*_*u*_^(*E*)^ and *p*_*u*_^(*I*)^ of users and the explicit-implicit feedback latent feature vectors *q*_*i*_^(*E*)^ and *q*_*i*_^(*I*)^ of items, and the explicit-implicit feedback vectors are added to complement each other to obtain the latent feature vectors *P*=[*p*_*i*_^(*E*)^ ⊕ *p*_*i*_^(*I*)^] of the user and the latent feature vectors *Q*=[*q*_*i*_^(*E*)^ ⊕ *q*_*i*_^(*I*)^] of the item, as the input of the training layer of the hybrid model of the EINMF algorithm. Through the training of the hybrid model layer, the output gets the shallow linear features of user preferences and the deep nonlinear features of user preferences and connects the two vectors to predict the degree of user preferences for noninteracted items and utilizes a new loss function based on explicit and implicit feedback proposed in this paper and the forward and backward propagation of the neural network model to update the relevant parameters of the EINMF model. Finally, the user's top-N personalized item recommendation list is gained by predicting the user's preference value for the item that is most similar to the actual preference based on the optimal parameters. The overall framework of the EINMF model is shown in Figure 1.

Through the hybrid model layer, based on the matrix decomposition concept, with the user and item latent feature vectors as inputs, the shallow linear preference features of the user are obtained using the dot product operation, as shown in formula (9), where symbol ⊙ refers to the product of the corresponding elements of the two vectors (i.e., dot product):

(9) $$\begin{array}{c} \phi_{\mathrm{EI}}^{\mathrm{dot}}\left(P_{u},Q_{i}\right)=P_{u}⊙Q_{i}. \end{array}$$

In the neural network model, the user latent feature vector and the item latent feature vector are connected together, utilizing the hidden layer of the multilayer perceptron (MLP) to obtain the deep nonlinear preference features of the user and the item to model the complex relationship between the user and the item; to obtain the multilayer nonlinear projection of the user-item interaction, the multilayer complex user preference features in the hybrid model layer of EINMF are defined as follows:

(10) $$\begin{array}{c} \begin{array}{c} z_{\mathrm{EI}}=\left[\begin{array}{c} P_{u}\\ Q_{i} \end{array}\right]\\ \varphi_{\mathrm{EI}}^{1}\left(z_{\mathrm{EI}}\right)=a_{\mathrm{EI}}^{1}\left(W_{\mathrm{EI}}^{1}z_{\mathrm{EI}}+b_{\mathrm{EI}}^{1}\right)\\ \varphi_{\mathrm{EI}}^{2}\left(z_{\mathrm{EI}}^{1}\right)=a_{\mathrm{EI}}^{2}\left(W_{\mathrm{EI}}^{2}z_{\mathrm{EI}}^{1}+b_{\mathrm{EI}}^{2}\right)\\ \vdots \\ \varphi_{\mathrm{EI}}^{X}\left(z_{\mathrm{EI}}^{X-1}\right)=a_{\mathrm{EI}}^{X}\left(W_{\mathrm{EI}}^{X}z_{\mathrm{EI}}^{X-1}+b_{\mathrm{EI}}^{X}\right) \end{array}, \end{array}$$

where *W*_EI_^*X*^, *b*_EI_^*X*^, and *a*_EI_^*X*^ denote the weight matrix, bias vector, and activation function of the *X*-th layer of the multilayer perceptron, respectively. Here, we use ReLU as the activation function because it has been shown to be more expressive than other functions and can effectively handle the gradient disappearance problem [13, 20]. *X* denotes the number of layers in the multilayer perceptron (MLP).

The output of the prediction layer of the EINMF model is the preference prediction value of the interaction between user *u* and item *i* based on explicit and implicit feedback, connecting the user linear and nonlinear preference features, and the preference prediction formula is defined as follows: *h*^*T*^ denotes the weight parameter of the user prediction layer and *a*_out_ denotes the activation function of the prediction layer, using the Sigmoid function as the activation function of the output layer, and the output prediction value is between 0 and 1, which can well combine linear features and nonlinear features. The user preference prediction function is defined as follows:

(11) $$\begin{array}{c} {\hat{y}}_{ui}^{\left(\mathrm{EI}\right)}=a_{\mathrm{out}}\left(h^{T}\left[\begin{array}{c} \varphi_{\mathrm{EI}}^{\mathrm{dot}}\\ \varphi_{\mathrm{EI}}^{\mathrm{MLP}} \end{array}\right]\right). \end{array}$$

### 3.3. EINMF Model Loss Function

The loss function is a crucial part of recommendation model construction which concerns the performance of the recommendation algorithm. It is essentially an objective optimization function of the recommendation model, which can be defined based on both explicit and implicit feedback data of user-item interaction. This paper proposes a new loss function based on the point-by-point loss function, that is, a hybrid explicit-implicit feedback loss function, which aims to obtain accurate ratings with a view to being more applicable to predicting accurate user preferences. At present, the commonly used point-by-point loss functions in recommendation algorithms are mainly the squared loss function and the binary cross-entropy loss function.

The square loss function has been applied in many matrix factorization recommendation algorithms [3, 6, 11, 17], but the square loss is used with the following assumptions: the predicted values are generated from Gaussian distribution, which is less consistent with the binary value distribution of the implicit feedback [4]. Therefore, the square loss function is better used in matrix factorization recommendation algorithms based on explicit feedback than matrix factorization recommendation algorithms based on implicit feedback. The basic definition of the squared loss function is shown in the following formula:

(12) $$\begin{array}{c} L_{squ}=\underset{u\in U}{\sum }\underset{i\in I}{\sum }w_{ui}{\left(y_{ui}-{\hat{y}}_{ui}\right)}^{2}. \end{array}$$

For the implicit feedback, based on the characteristics of implicit feedback binarization, subsequent researchers proposed a point-by-point loss function based on the binary classification optimization task [13, 20], named the binary cross-entropy loss function, which performs better than the squared loss with the implicit feedback recommendation algorithm. The basic definition of the binary cross-entropy loss function is shown in the following formula:

(13) $$\begin{array}{c} L_{\log}=-\underset{u\in U}{\sum }\underset{i\in I}{\sum }y_{ui}\log\;{\hat{y}}_{ui}+\left(1-y_{ui}\right)\log\left(1-{\hat{y}}_{ui}\right). \end{array}$$

DMF [20] applied the cross-entropy loss function to the normalized explicit ratings and proved its effectiveness for the optimization of the recommendation model parameters. Therefore, in this paper, a new loss function is designed to normalize the explicit ratings to values between 0 and 1, which can be made suitable for the application of the cross-entropy loss function. Since both explicit user rating data and implicit feedback data reflect user preferences [1], we use both explicit feedback and implicit feedback information together in the objective optimization function to optimize and update the recommendation model parameters. The explicit rating normalized loss function is defined as in formula (14), and the implicit feedback binary loss function is defined as in formula (15).

(14) $$\begin{array}{c} L_{E}=-\underset{u\in U_{E}}{\sum }\underset{i\in I_{E}}{\sum }\frac{r_{ui}}{Max\left(R\right)}\log\;{\hat{y}}_{ui}+\left(1-\frac{r_{ui}}{Max\left(R\right)}\right)\log\left(1-{\hat{y}}_{ui}\right). \end{array}$$

In formula (14), Max(*R*) represents the maximum explicit user rating of items in the training set, which is used in this paper to normalize the user-item ratings; for example, in the 5-point rating dataset, if the user rating of items is 3 and the maximum rating in the training set is 5, the normalized rating value can be obtained as 3/5 = 0.6. Thus, different rating values have different effects on the loss.

(15) $$\begin{array}{c} L_{I}=-\underset{u\in U_{I}}{\sum }\underset{i\in I_{I}}{\sum }ir_{ui}\log\;{\hat{y}}_{ui}+\left(1-ir_{ui}\right)\log\left(1-{\hat{y}}_{ui}\right). \end{array}$$

In formula (15), *ir*_*ui*_ denotes the binarized implicit rating of the implicit feedback of user *u* to item *i*. Different types of feedback data have their own suitable loss functions, and the recommendation model in this paper utilizes two types of data for model construction and training. Therefore, the two loss functions are combined as the loss function of the EINMF, and the loss function is defined as formula (16), giving them different weights, making full use of the respective characteristics of the explicit and implicit feedback data. This new loss function is named the explicit-implicit feedback hybrid loss function, and *η* is used to control the respective weights of the explicit-implicit feedback losses in the loss function.

(16) $$\begin{array}{c} L=\eta L_{I}+\left(1-\eta \right)L_{E}. \end{array}$$

## 4. Experiments

Various experiments were designed on real-world open-source MovieLens datasets to verify the feasibility, effectiveness, and robustness of the EINMF user preference prediction algorithm and the new loss function.

### 4.1. Dataset

We evaluate the EINMF model and the baseline model with two widely adopted datasets in the field of recommender systems, MovieLens-100K (ml-100k) and MovieLens-1M (ml-1m); MovieLens contains multiple rating datasets collected from the MovieLens website over different time periods. MovieLens-100K (ml-100k) contains 100,000 ratings for 1682 movies from 943 users, and MovieLens-1M (ml-1m) contains over 1 million ratings for 3706 movies from 6040 users. The ml-100k dataset is not preprocessed in this paper because it is already filtered. Preprocessing is only done in the ml-1m dataset before the experiment, and users with less than 10 ratings and items with less than 10 ratings are filtered and removed to exclude the interference of abnormal cold data.

Table 2 presents the specific statistics of the two datasets after preprocessing.

### 4.2. Evaluation Methods

In this paper, the cross-validation method [13, 15, 20], which is widely used in deep learning recommendation models, is used to evaluate the performance of the recommendation system. The rating dataset is split into training data, validation data, and test data with the split ratio of [0.8, 0.1, 0.1]. First, the scoring dataset was cross-sectioned into 10 sets of the same size, and then 8 sets were used as training data for building the recommendation model, 1 set was used as validation set for model parameter tuning, and finally 1 set was used as test set for testing the accuracy and robustness of the final model.

Most recommendation systems utilize error loss assessment methods such as root mean square error (RMSE) and mean absolute error (MAE) to assess the similarity between users' predicted preferences and true preferences, but top-N based recommendation tasks, which recommend the top-N list of most interesting items for users based on their ranking of item preference predictions, use a ranking assessment compared to error loss method which is more realistic [21]. Therefore, we adopt hit rate (HR) and normalized discounted cumulative gain (NDCG) based on ranking performance evaluation for deep learning recommendation model performance [13, 20]. In this paper, the recommended item list generated by the predicted ranking of user preferences is defined as *Re*_*u*_={*re*_*u*_^1^, *re*_*u*_^2^,…, *re*_*u*_^*N*^}, where *N* represents the length of the recommendation list, that is, the number of items in the recommendation list, and *re*_*u*_^*i*^ represents the *i*-th position of the item ranked in the *Re*_*u*_ list according to the predicted preference value; the set of items interacted by user *u* in the validation set and the test set is defined as *I*_*u*_. For both evaluation methods, larger values represent better performance of the recommendation system, and the two evaluation methods are calculated as follows.

Hit rate: it is used to evaluate the accuracy of the recommendation system, that is, whether the test items are included in the top-N item recommendation list; the HR calculation is shown in formula (17), where |*U*| indicates the number of users in the validation set and the test set.
  (17) HR@N=∑uReu∩IuU.
Normalized discounted cumulative gain (NDCG): Used to measure the ranking accuracy of the recommendation system, that is, whether the test item is ranked at the top of the top-N item recommendation list; the NDCG calculation is shown in the following formula:

(18) $$\begin{array}{c} \mathrm{NDCG}@N=\frac{1}{Z}\mathrm{DCG}@N=\frac{1}{Z}\frac{1}{\left|U\right|}\overset{U}{\underset{u}{\sum }}\overset{N}{\underset{i=1}{\sum }}\frac{2^{rel_{u}^{i}}-1}{\log_{2}\left(i+1\right)}, \end{array}$$

where *Z* is the normalization constant and is the approximate maximum value of DCG@*N*. At this point, |*U*| denotes the number of users in the validation set and the test set, and *i* denotes the ranking of the item in the recommendation list. *rel*_*u*_^*i*^ denotes the true relevance of user *u* to the item at the *i*-th ranking position in the recommendation list, which is 1 if there is interaction between them; otherwise, it is 0.

### 4.3. Baselines and Experiment Parameters

In this paper, the EINMF recommendation algorithm is compared with the five following baseline algorithms:

  Pop [2]: a typical recommendation method that ranks items by their popularity based on the number of interactions and is a nonpersonalized recommendation method used as a baseline evaluation comparison for personalized recommendation methods.
  Item-KNN [5]: a standard item-based collaborative filtering method for measuring the similarity among items to achieve personalized recommendations as a baseline approach.
  BPR-MF [10]: a pairwise ranking method, which optimizes the recommendation method of MF model based on implicit feedback by pairwise Bayesian personalized ranking loss function, in order to learn from implicit feedback data, and is a common baseline for personalized recommendation of items.
  NCF [13]: an advanced neural network-based collaborative filtering method that uses a multilayer perceptron to obtain nonlinear information about user-item interactions and optimizes model parameters, using binary cross-entropy loss. For a fair comparison, the experiments use the same embedding size, number of hidden layers, and predictor size for both NCF and EINMF models.
  DMF [20]: a deep learning recommendation method using a multilayer perceptron for rating matrix factorization, where the latent factors of users and items are trained by a multilayer perceptron to obtain predicted values of user preferences which are most similar to the true user-item ratings.

#### 4.3.1. Experiment Parameters Setting

The experiments in this paper are based on Python 3.7, Keras 2.4.3, and PyTorch 1.7.1 to complete the comparison experiments among EINMF and other baselines. Relevant parameters are set as follows: the maximum number of model training iterations is set to 100, and the training is stopped early when the evaluation value of the validation set no longer has growth at 10 iterations. For the neural network, we used a Gaussian distribution (mean of 0 and standard deviation of 0.01) to randomly initialize the model parameters; a small-batch Adam optimizer was used for optimization of the model parameters with a training batch size of 1024. The learning rate of BPR-MF was set to 0.001 and the number of negative samples was 4.

For the deep learning baselines NCF and DMF, the learning rate of NCF model is set to 0.001 and the negative sampling value is set to 4 according to the optimal results described in literature [13, 20] and the actual result; the learning rate of DMF model is set to 0.001 when training on the ml-100k dataset and 0.0005 when training on the ml-1m dataset, and the negative sampling values are both set to 2. The learning rate of the EINMF is set to 0.0001, and the discard rate parameter is also added [22], which randomly discards some neurons during the training of the neural network, for preventing the model overfitting during training, thus causing a large deviation between the test set evaluation results and the validation set evaluation results that increase the generalizability of the model, with a discard rate of 0.2. The comparison of the EINMF explicit and implicit loss function weight parameter and the influence of important parameters such as the number of neural network layers on the performance of the EINMF model will be specifically analyzed in the experiments.

### 4.4. Performance Comparison

Table 3 shows the results of the comparative analysis of the five baselines and the EINMF based on the performance evaluation metrics HR and NDCG of the top-N task for ranked recommendations on two datasets that have different sparsity and different sizes. For a fair comparison, the embedding dimensions of both users and items for the embedding-based methods, the BPR-MF, NCF, DMF, and the EINMF, proposed in this paper are set to 64. In addition, since the difference in the predicted number of recommended items *N* also has an impact on the performance of the recommendation system, the evaluation metrics of each recommendation model with the number of recommended items *N* ∈ {5,10,20} are tested to increase the diversity of top-N task evaluation.

As shown in Table 3, the values of hit rate (HR) and normalized discounted cumulative gain (NDCG) of the EINMF and baselines gradually increase as the number *N* of top-N task recommendation lists increases, which is consistent with the actual recommendation requirements and indicates that the recommendation algorithms are realistic. On the ml-100k dataset, the hit rate (HR@N) of the EINMF model improved by a minimum of 1.71%, a maximum of 8.05%, and an average of 4.87% compared to the best baseline, while the NDCG@N improved by a minimum of 10%, a maximum of 18.38%, and an average of 14.41% compared to the best baseline; on the ml-1m dataset, the hit rate (HR@N) of the EINMF model improved by a minimum of 4.23%, a maximum of 8.46%, and an average of 6.53% compared to the best baseline, while the NDCG@N improved by a minimum of 7.46%, a maximum of 11.88%, and an average of 9.53% compared to the best baseline.

In summary, it is shown that the recommendation accuracy of EINMF algorithm for different top-N tasks of two datasets with different sparsity and data size is better than those of the baselines and latest deep learning recommendation algorithms, which effectively improves the accuracy of recommendation system recommendations and corresponds to the needs of real recommendation scenarios.

In the deep learning matrix factorization recommendation algorithm, the size of embedding dimension is one of the important factors affecting the performance of the recommendation model, and the parameter embedding-dim denotes the dimension of user vector and item vector.

**Figure 2:**
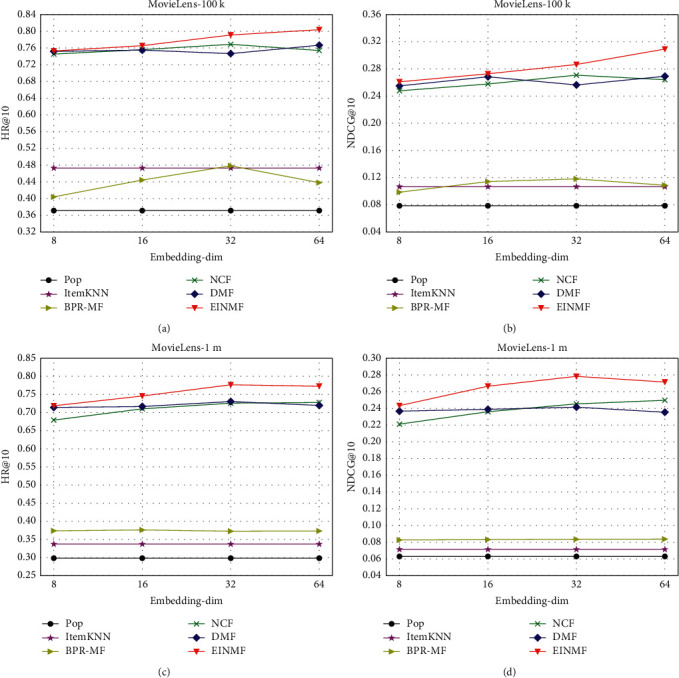
Evaluation results of different embedding dimensions of each model. (a) MovieLens-100k-HR@10, (b) MovieLens-100k-NDCG@10, (c) MovieLens-1m-HR@10, and (d) MovieLens-1m-NDCG@10.

As the analysis in Figure 2 shows, for the two ranking metrics HR@10 and NDCG@10, the EINMF proposed in this paper outperforms other baselines in terms of evaluation values on two datasets with different sparsity and size as well as on different embedding dimensions. In addition, as shown in Figure 2, the performance of the recommendation system improves as the latent factor embedding dimension increases, indicating that a larger dimension captures more hidden information about users and items, which helps to enhance the modeling capability. However, as shown in Figures 2(c) and 2(d), the performance of the recommendation system starts to degrade when the latent factor embedding dimension is too large. Therefore, choosing the appropriate embedding dimension based on the characteristics of the dataset and so forth is critical to the performance improvement and training prediction speed effect of the recommendation system. As shown in Figure 2, the evaluation results of all models on two datasets, ml-100k and ml-1m, show that the recommendation performance of the EINMF with latent factor embedding dimension 16 is even higher than the best baseline embedding dimension of 64, indicating that the EINMF is very effective for the performance improvement of the recommendation system. As shown in Figures 2(a) and 2(b), the evaluation comparison results of all models on the ml-100k dataset show that the recommendation performance of EINMF with latent factor embedding dimension of 8 is better than optimal embedding dimension evaluation metrics of the best baseline. The above analysis shows that the EINMF proposed in this paper greatly outperforms both the classical model and the state-of-the-art deep learning models NCF and DMF in recommendation performance and stability, which proves the effectiveness of the EINMF for top-N recommendation tasks.

### 4.5. Impact of Different Parameters

**Figure 3:**
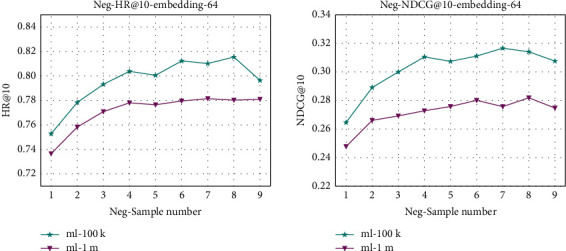
Performance of EINMF with regard to different negative sample numbers on the two datasets. (a) Neg-HR@10-embedding-64. (b) Neg-NDCG@10-embedding-64.

Negative sample number, that is, a certain number of randomly selected items from the items where users do not interact with the items, is essential for the performance improvement of the recommender system. To analyze the effect of negative sample number on the performance of the recommendation system, we set the number of negative samples, neg-num ∈ [1, 2, 3, 4, 5, 6, 7, 8, 9], on two datasets, and the comparison of the experimental results is shown in Figure 3.

From the analysis of Figure 3, we can see that the performance of the recommendation system gradually improves with the increase of the number of negative samples, but the performance of the recommendation system decreases when the number of negative samples is too many. The optimal number of negative samples for this model is in the range of 4–8, but the increase in the number of negative samples will lead to an increase of training parameters, resulting in an increase in the time for one training iteration, such as the average training time of the EINMF model with the negative sample number 8 is 2.5 times higher than that with the negative sample number 4. The personalized recommendation system requires high timeliness in large dataset training and needs to be adjusted in time with the change of user preferences. Therefore, too much time spent can cause a lag in the actual recommendation which leads to inaccurate recommendation results. From the results of the evaluation metrics of the above two datasets, we can see that the optimal negative sampling range of the EINMF is in the range of 4–6.

#### 4.5.1. Different Loss Function Weight

**Figure 4:**
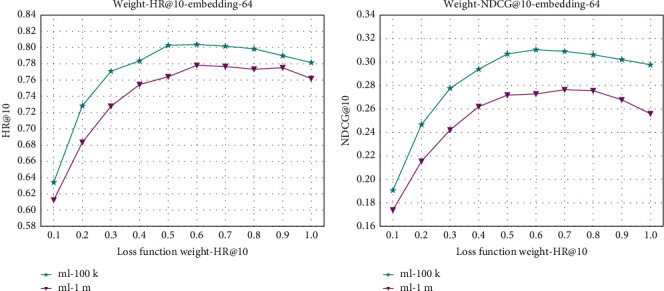
Performance of EINMF with regard to different loss function weight on the two datasets. (a) Weight-HR@10-embedding-64. (b) Weight-NDCG@10-embedding-64.

For loss function weight (*η*), in this paper, a new loss function is proposed in which the explicit-implicit feedback is incorporated into the loss function (i.e., the objective optimization function) by different weights for the optimization of the EINMF, and the values of the explicit-implicit feedback loss function with different proportional weights may have different effects on the recommended performance, and this paper sets the range of *η*∈ [0.1, 1.0] with a step size of 0.1. A comparison of the evaluation results with different weights is shown in Figure 4.

As shown by the explicit-implicit feedback hybrid loss function formula (16), when weight *η* takes the value of 0, the new objective optimization function proposed in this paper is a pure explicit-feedback-based loss function, and when weight *η* is 1, the new objective optimization function is a pure implicit-feedback-based loss function. As can be seen from Figure 4, when weight *η* is 0, the performance of the recommendation effect should be the worst; with the increase of weight *η*, the performance of the recommendation system improves rapidly, and the performance of the recommendation system reaches the best between the weights of 0.5–0.7; the change is generally, and after 0.7, all the evaluation metrics start to decline rapidly. From the above analysis, it can be seen that the EINMF model reaches the best performance when the weight of the hybrid loss function is about 0.6, and the new hybrid loss function proposed in this paper is very effective in optimizing the performance of the recommender system.

## 5. Summary and Outlook

To improve the accuracy of recommendation systems and enhance user satisfaction, this paper proposes a user preference prediction neural matrix factorization algorithm integrating explicit feedback and implicit feedback. The matrix factorization algorithm is used to mine the shallow linear features of explicit-implicit feedback of user-project interaction and the deep nonlinear features of explicit-implicit feedback using neural networks, make full use of the complementarity of explicit-implicit feedback, and solve the defects of the current deep learning algorithm in training the model using only one feedback data. In addition, according to the construction requirements of the model integrating explicit-implicit feedback for the neural matrix factorization model, a hybrid loss function integrating explicit-implicit feedback is proposed for the optimization of model parameters, which improves the accuracy of the recommendation system for user preference prediction. The experimental results demonstrate the effectiveness and robustness of the EINMF algorithm. As a kind of collaborative filtering algorithm, EINMF algorithm builds a recommendation system based on user history data. In the future, it will consider integrating user and project attribute data and more types of explicit-implicit feedback data including comments and clicks into the model or adopt a better explicit-implicit feedback data fusion method to further alleviate the sparse data and cold start problems of collaborative filtering algorithm.

## Figures and Tables

**Table 1:** Symbol definition.

Symbol	Description
*U*	Set of users
*V*	Set of items
*R*=[*r*_*ui*_]_*m*×*n*_	User-item rating matrix
*p*_*u*_^(*E*)^	Explicit feedback feature vector of user *u*
*q*_*i*_^(*E*)^	Explicit feedback feature vector of item *i*
*IR*=[*ir*_*ui*_]_*m*×*n*_	User-item implicit interaction matrix
*p*_*u*_^(*I*)^	Implicit feedback feature vector of user *u*
*q*_*i*_^(*I*)^	Implicit feedback feature vector of item *i*
*P*_*u*_	User's latent factor vector matrix
*Q*_*i*_	Item's latent factor vector matrix
*a*_*EI*_^X^	Activation function of the *X*-th layer in the neural network
W_*EI*_^X^	Weight matrix of the *X*-th layer in the neural network
b_*EI*_^X^	Deviation value of the *X*-th layer in the neural network
*a*_out_	Activation function of the prediction layer
${\hat{y}}_{ui}^{\left(\text{EI}\right)}$	Preference prediction for the interaction between user *u* and item *i*
Θ	Set of parameters related to the neural network
*η*	Controlling the weight of explicit and implicit feedback in the loss calculation

**Table 2:** Statistics of the datasets.

Dataset	Rating	User	Item	Density	Rating range
ml-100k	100,000	943	1,682	93.70%	[1, 5]
ml-1m	1,000,209	6,040	3,260	94.93%	[1, 5]

**Table 3:** Performance of EINMF compared with other algorithms (embedding size = 64).

Dataset	MovieLens-100k						MovieLens-1m					
	HR@*N*			NDCG@*N*			HR@*N*			NDCG@*N*		
Model	*N* = 5	*N* = 10	*N* = 20	*N* = 5	*N* = 10	*N* = 20	*N* = 5	*N* = 10	*N* = 20	*N* = 5	*N* = 10	*N* = 20
Pop [2]	0.2031	0.3712	0.4761	0.0718	0.0786	0.0863	0.2015	0.2983	0.4228	0.0718	0.0786	0.0863
Item-KNN [5]	0.3160	0.4730	0.5758	0.0976	0.1067	0.1185	0.2237	0.3371	0.4874	0.0677	0.0714	0.0817
BPR-MF [10]	0.2874	0.4380	0.5822	0.0971	0.1088	0.1277	0.3340	0.4804	0.6267	0.1135	0.1206	0.1409
NCF [13]	0.6002	0.7540	0.8367	0.2662	0.2641	0.2890	0.603	0.7278	0.8268	0.2608	0.2497	0.2525
DMF [20]	0.6458	0.7667	0.8738	0.2672	0.2692	0.2835	0.5892	0.7197	0.8257	0.2401	0.2354	0.2413
EINMF	**0.6978**	**0.8038**	**0.8887**	**0.3163**	**0.3092**	**0.3179**	**0.6540**	**0.7781**	**0.8618**	**0.2880**	**0.2728**	**0.2825**
MI (%)	**8.05**	**4.84**	**1.71**	**18.38**	**14.86**	**10.00**	**8.46**	**6.91**	**4.23**	**7.46**	**9.25**	**11.88**

“MI” indicates the smallest improvements of our EINMF over the corresponding baseline. The optimal value of each metric of the baseline top-N task is underlined in the table.

## Data Availability

The MovieLens datasets used to support the findings of this study have been opened on the Internet. Copies of these data can be obtained free of charge from https://grouplens.org/datasets/movielens/.
